# Live Cell Therapy as Potential Risk Factor for Q Fever

**DOI:** 10.3201/eid2307.161693

**Published:** 2017-07

**Authors:** Maja George, Andreas Reich, Klaus Cussler, Herrmann Jehl, Florian Burckhardt

**Affiliations:** Author affiliations: Robert Koch Institute, Berlin, Germany (M. George);; European Centre for Disease Prevention and Control, Stockholm, Sweden (M. George);; Institute for Infectious Disease Prevention Landau, Landau, Germany (M. George, F. Burckhardt);; Bavarian Health and Food Safety Authority, Oberschleissheim, Germany (A. Reich);; Paul Ehrlich Institut, Langen, Germany (K. Cussler);; Health Department of the Bad Duerckheim District, Bad Duerkheim, Germany (H. Jehl)

**Keywords:** live cell therapy, Q fever, Coxiella burnetii, bacteria, risk factor, outbreaks, inhalation exposure, intramuscular injection, sheep, fetal tissue, occupational exposure, zoonoses, Canada, Germany

## Abstract

During an outbreak of Q fever in Germany, we identified an infected sheep flock from which animals were routinely used as a source for life cell therapy (LCT), the injection of fetal cells or cell extracts from sheep into humans. Q fever developed in 7 LCT recipients from Canada, Germany, and the United States.

Gram-negative intracellular bacteria (*Coxiella burnetii*) cause Q fever, a zoonotic disease usually subclinical in livestock and humans. Typically, human patients show signs and symptoms, such as fever, severe headache, nausea, pneumonia, or hepatitis, 2−3 weeks after infection. Chronic Q fever develops in ≈1%–5% of patients (*1*).

On August 5, 2014, a local health department in the Federal State of the Rhineland Palatinate in southern Germany alerted the Federal State Agency for Consumer and Health Protection (FSACHP) (Landau, Germany) after detecting a cluster of 8 patients with pneumonia in a rural community during a 6-week period. The local health department and FSACHP started a joint outbreak investigation to identify cases, find the source of the outbreak, and stop disease transmission.

On August 12, five of 8 patients tested by ELISA and immunofluorescence test (IFT) by the local health department had results consistent with acute *C. burnetii* infection. The local health department issued a public health warning in the local media and advised anyone in the affected community with influenza-like symptoms or pneumonia in the past 4 months to be tested for Q fever by their general practitioner. In addition, the department offered free testing to pregnant women and persons with cardiovascular risk factors (e.g., heart valve defects), irrespective of symptoms (*2*).

Case-patients were defined as persons who had phase II IgM or IgG titers for Q fever by ELISA or IFT in 2014. Data for these case-patients were entered into the German Electronic Surveillance System for Infectious Disease Outbreaks (*3*). Thirteen residential case-patients (6 men, 7 women) who lived in the affected county (Bad Duerkheim) were identified; 11 reported symptoms compatible with Q fever, of whom 6 were hospitalized (Figure). Median age for residential case-patients was 50 (range 32–59) years for women and 44 (range 26–56) years for men.

**Figure:**
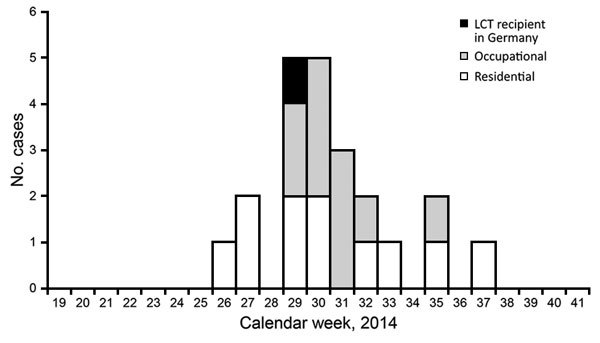
Residential (n = 11), occupational (n = 10), and recipient (n = 1) cases of Q fever related to live cell therapy (LCT), by week of symptom onset compatible with Q fever, Rhineland-Palatinate, Germany, 2014.

Because all residential case-patients lived within 1.5 km of a flock of 1,000 sheep, the FSACHP tested random samples from these sheep. Of 61 sheep tested, 25 were positive for *C. burnetii* by ELISA of serum samples and 2 by PCR of vaginal swab specimens. During the investigation, the local health department discovered that young rams and pregnant ewes in the flock had been used as donor animals for live cell therapy (LCT) at 2 medical facilities in a district 10 km from the farm. LCT is injection of fetal cells or cell extracts from sheep into humans. The flock was banned for LCT production, and veterinary control measures (e.g., indoor housing and immunization of sheep) were initiated to stop transmission.

Staff members of LCT facilities were offered serologic testing by ELISA or IFT. Sixteen persons with occupational cases (3 men, 13 women) were reported; 10 showed onset of disease, and 2 were hospitalized (Figure). Median age for occupational case-patients was 48 (range 28–62) years for women and 45 (range 35–50) years for men.

LCT is an alternative treatment (without medical evidence of effectiveness) that is marketed worldwide online. It consists of intramuscular injections of cell suspensions from fetal sheep to human recipients for rejuvenation (anti-aging) and other ailments. Apart from national recipients, medical tourists from North America and Asia travel to Germany to receive injections. In August, the FSACHP was notified of a patient from Canada who received LCT injections on May 28, 2014, and became ill in June, before *C. burnetii* was detected in the asymptomatic donor sheep herd (*4**,**5*).

Newspaper coverage in October of the Q fever outbreak and the potential link to the LCT recipient from Canada alerted an LCT recipient in Germany who had recovered from a previously unidentified illness after receiving LCT in Rhineland Palatinate (*4**,**6*). The patient became ill (fever, severe diarrhea, and fatigue) 1 day after receiving LCT injections on July 14, lost 9 kg, and was hospitalized for 10 days. She was positive for Q fever by IFT in October 2014 and had a phase II IgG titer (1:65,536) that was higher than her phase I IgG titer (1:4,096), which indicated a recent infection. She had no other contact with sheep or sheep products during her stay at the LCT facility or thereafter.

A pharmacovigilance report by the Paul Ehrlich Institut (Langen, Germany) indicated that LCT treatment was the probable cause of the Q fever (*7*). The county ordered both LCT facilities to advise all 830 LCT recipients treated since January 2014 to consult their general practitioner about their possible risk for Q fever. This advice prompted 5 US citizens who received injections on May 30 in one of the clinics to be tested for Q fever. An investigation by the US Centers for Disease Control and Prevention (Atlanta, GA, USA) identified recent Q fever in all 5 patients (phase II IgG titers <1:65,536 at 2–6 months postinjection) (*5*).

Several facilities offer LCT in Germany, although the federal ministry of health recently released an assessment stating that the use of LCT is unsafe (*8*). Therefore, practitioners worldwide should be informed that working at an LCT clinic or receiving LCT injections should be considered potential risk factors for Q fever.
